# Current updates on the role of reactive oxygen species in bladder cancer pathogenesis and therapeutics

**DOI:** 10.1007/s12094-020-02330-w

**Published:** 2020-03-18

**Authors:** D. Liu, X. Qiu, X. Xiong, X. Chen, F. Pan

**Affiliations:** 1 Department of Urology, Union Hospital, Tongji Medical College, Huazhong University of Science and Technology, Wuhan, 430022 China; 2 Fudan University Shanghai Cancer Center, Department of Oncology, Shanghai Medical College, Fudan University, Shanghai, 200032 China; 3 State Key Laboratory of Oncology in South China, Collaborative Innovation Center for Cancer Medicine, Sun Yat-Sen University Cancer Center, Guangzhou, 510060 China; 4 Department of Pathophysiology, School of Basic Medicine, Tongji Medical College, Institute of Brain Research, Key Laboratory of Neurological Diseases, Ministry of Education, Hubei Provincial Key Laboratory of Neurological Diseases, Huazhong University of Science and Technology, Wuhan, 430030 China

**Keywords:** Reactive oxygen species, Oxidative stress, Bladder cancer, Apoptosis, Therapeutics

## Abstract

Bladder cancer (BCa) is the fourth most common urological malignancy in the world, it has become the costliest cancer to manage due to its high rate of recurrence and lack of effective treatment modalities. As a natural byproduct of cellular metabolism, reactive oxygen species (ROS) have an important role in cell signaling and homeostasis. Although up-regulation of ROS is known to induce tumorigenesis, growing evidence suggests a number of agents that can selectively kill cancer cells through ROS induction. In particular, accumulation of ROS results in oxidative stress-induced apoptosis in cancer cells. So, ROS is a double-edged sword. A modest level of ROS is required for cancer cells to survive, whereas excessive levels kill them. This review summarizes the up-to-date findings of oxidative stress-regulated signaling pathways and transcription factors involved in the etiology and progression of BCa and explores the possible therapeutic implications of ROS regulators as therapeutic agents for BCa.

## Background

Bladder cancer (BCa) is a common urinary malignancy which ranks approximately ninth among the most commonly diagnosed cancers and is the thirteenth most common cause of cancer death worldwide [1]. Annually, more than 12 million new cases occur throughout the world, with 5.4 million in developed countries and 6.7 million in developing countries [2]. In 2016, there were 76,960 newly diagnosed BCa cases and 16,390 deaths in the United States alone [3]. The diagnosis of BCa is 3 to 4 times more common in men than in women [4]. Unlike many other cancers (e. g., breast cancer), BCa survival rates have not improved in more than three decades [5]. Treating BCa is problematic due to high cost, multicentric occurrence, and a high recurrence rate [6, 7]. Following approximately 1 year of platinum-based chemotherapy, patients with newly diagnosed advanced or metastatic BCa have a five-year overall survival rate of merely 6% [8]. Therefore, it is imperative to develop an effective treatment strategy for BCa to reduce recurrence rate, minimize adverse effects, and improve overall survival rates.

Reactive oxygen species (ROS) exist as a variety of oxygen-containing chemical metabolites. Normally, cells manage to maintain cellular redox homeostasis. Either excessive generation of ROS or an imbalance in the antioxidant defense system may increase ROS levels. As a result, oxidative stress may play an important role in the initiation and development of numerous diseases, including diabetes, hypertension, and cancer [9]. Mounting evidences show that antineoplastic agents can induce oxidative stress-associated apoptosis in cancer cells, which demonstrate a beneficial role for ROS.

ROS may exhibit a dual role in promoting or suppressing cancer formation. The purpose of this review is to summarize the causes of oxidative stress, evaluate its potential role in the etiology and progression of BCa, and explore the possibility of ROS regulators as tumor suppressors.

## Regulation of ROS in BCa cells

ROS is composed of both free radical and non-free radical oxygen intermediates, such as hydrogen peroxide (H_2_O_2_), superoxide (O_2_^**.**−^), singlet oxygen (^1^O_2_), and hydroxyl radical (·OH), which can be induced from both endogenous and exogenous sources [10]. Intracellular ROS are mainly produced from the mitochondrial respiratory chain. During aerobic respiration, the electron transport chain passes electrons to oxygen (O_2_), reducing oxygen to H_2_O. Around 2–3% of electrons directly leak from respiratory complex I and III, reducing oxygen to form a number of free radicals, such as O_2_^**.**−^, ·OH^−^, and H_2_O_2_. Induction of ROS could also be executed by cytochrome P450 in the endoplasmic reticulum and numerous enzyme systems, including intracellular lipoxygenase, cyclooxygenase, and xanthine/hypoxanthine. Exogenous sources of ROS can be produced from environmental agents, including non-genotoxic carcinogens, chlorinated compounds, radiation, metal ions, barbiturates, phorbol esters, and several peroxisome-proliferating compounds [11]. Smoking and exposure to industrial chemicals, like aromatic amine, are risk factors for BCa [12, 13]. *N*-nitrosodibutylamine (BBN), the main component of tobacco, has been considered to be a stimulus for intracellular ROS-induced oxidative stress and a carcinogen that initiates BCa in rodents [14].

Physiologically, cellular redox homeostasis is tightly maintained by an elaborate endogenous antioxidant defense system. This system contains endogenous antioxidant enzymes: superoxide dismutase (SOD), catalase and glutathione peroxidase (GPx), and glutathione (GSH), as well as low-molecular-weight scavengers, such as uric acid, coenzyme Q, and lipoic acid. GSH is a tripeptide composed of glutamic acid, cysteine, and glycine. GSH is a carrier of multiple thiol groups and is widely distributed throughout the human body. GSH exists both in reduced (GSH) and oxidized (GSSG) states. The GSH/GSSG balance is dynamically maintained. During oxidative stress, GSH is converted to its oxidized state, GSSG, which is an important oxidant for cells [15]. The antioxidant defense system promotes reduction of H_2_O_2_ and lipid peroxide and eliminates the O_2_^**.**−^ anion, preventing cell and tissue oxidative damage caused by a free radical chain reaction.

## ROS/oxidative stress-related signaling pathways in BCa

**Fig. 1:**
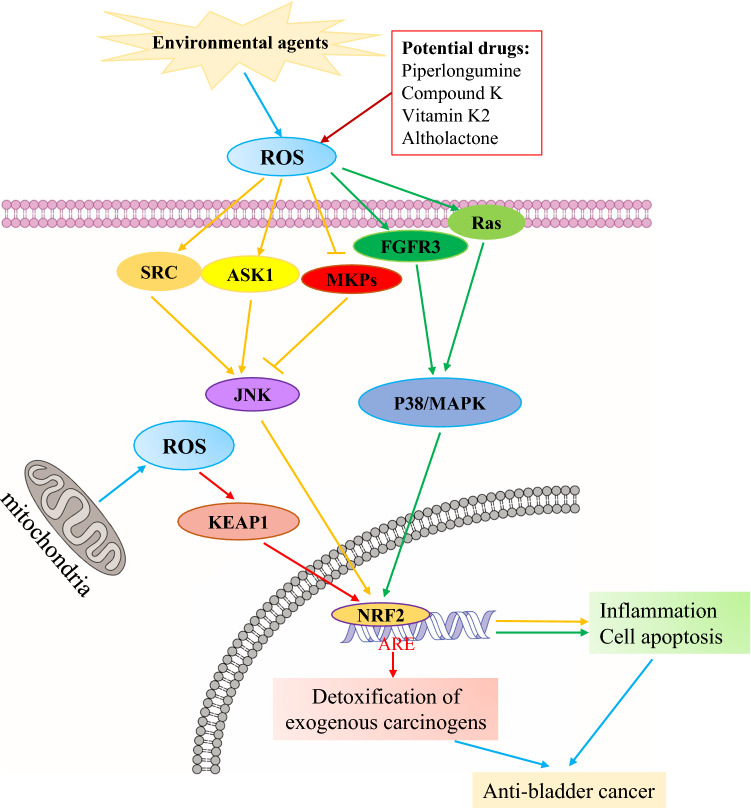
ROS/oxidative stress-related signaling pathways in BCa. ROS/oxidative stress from environmental agents leads to up-regulation/activation of SRC, ASK1, MKPs, FGFR3, and Ras which, in turn, activate JNK, p38/MAPK, KEAP1, and NRF2, either directly or indirectly. This further regulates downstream signaling proteins to promote or suppress BCa cell proliferation, migration, and invasion. In addition, the binding of ARE to NRF2 leads to activation of BCa cell apoptosis. → up-regulation/activation;  down-regulation/inhibition

As intracellular primary and secondary messengers, ROS participates in signal transduction and regulates cell proliferation, apoptosis, and tumorigenesis. ROS accomplishes this by modulating the expression of transcription factors, enzymes, and structural proteins [16–19]. ROS interacts with multiple signaling pathways to regulate the proliferation and apoptosis of cancer cells. For instance, ROS regulates both the extracellular signal-regulated kinase (ERK) signaling pathway and the AKT signaling pathway, which mediates ROS-activated programmed cell death. In particular, ROS-activated programmed cell death controls apoptosis-related proteins and causes oxidative damage in DNA to achieve oncogenic transformation [20–22]. Namely, ROS/MAPK and ROS/Keap1-Nrf2-ARE signaling pathways are two classic signaling pathways that are stimulated by ROS (Fig. 1).

### ROS/MAPK signaling pathway

As a type of cellular serine/threonine protein kinase, the mitogen-activated protein kinase (MAPK) signaling pathway controls diverse cellular reactions: cell proliferation, differentiation, transformation, and apoptosis. MAPK signaling pathway in mammals consists of 3 different signaling molecules: c-Jun N-terminal kinase (JNK), p38/MAPK, and ERK. When cells are stimulated by ultraviolet radiation, oxidative stress, and other stimuli, MAPK signaling pathway will be continuously activated. The overall effect of the MAPK signaling pathway is dependent on the duration of the stimulus. Transient activation of the MAPK signaling pathway is associated with gene expression, proliferation, and differentiation, whereas continuous activation of the MAPK signaling pathway induces cell death [23].

The JNK signaling pathway is important for cells to sense changes in their environments, and it is also involved in the regulation of cellular proliferation, differentiation, and apoptosis. With high bioactivity, ROS can be activated by stress stimulation, cytokines, and growth factors. After ROS activation, the JNK signaling pathway is initiated by ROS-stimulated signaling proteins, such as ASK1, SRC, and MKPs [24]. Activated ROS plays a critical role in mediating inflammation and cellular apoptosis through the JNK signaling pathway. Meanwhile, a positive feedback loop exists between the ROS and JNK signaling pathway, which implies that ROS production relies on the existence of JNK [25].

The p38/MAPK signaling pathway is vital for a cell stress reaction. The p38/MAPK signaling pathway is activated by stress and cytokines which participate in inflammation and immunoregulation. Furthermore, this pathway may trigger apoptosis and promote the repair of stress-damaged cells. Although isomerides of the p38/MAPK signaling pathway exist and are distributed with cell- and tissue-specificity, different p38/MAPK signaling pathways lead to different biological reactions in different cell types. It is generally believed that the p38/MAPK signaling pathway initiates the proliferation of urothelial cancer cells [26]. Moreover, a gene mutation in the MAPK signaling pathway has been regarded as one of the mechanisms for BCa. Both the frequent mutations in the fibroblast growth factor receptor 3 (FGFR3) and a mutation in the Ras gene have been found in superficial urothelial cancer [27].

### ROS/Keap1-Nrf2-ARE Pathway

Nuclear factor is a type of transcription factor that modulates the transcriptional activity of target genes through promoter binding. Nuclear factor kappa-light-chain-enhancer of activated B cells (NF-κB) is a major nuclear factor that participates in regulating a variety of biological functions in the human body. In 1991, H_2_O_2_ was found to activate NF-κB in melanoma cells cultured in vitro [28]. Subsequent research findings demonstrated that the activation of NF-κB by ROS was cell type-specific. For example, in epithelial cells, ROS initiated the NF-κB signaling pathway mainly by enhancing the expression of protein kinase D [28, 29]. While in LN229 cells, ROS inhibited the activation of NF-κB [30]. Furthermore, activation of NF-κB has been found to stimulate the generation of ROS, and antioxidants can effectively inhibit the activation of NF-κB caused by tumor necrosis factor [29].

The nuclear factor erythroid 2-related factor 2 (Nrf2) is a newly discovered nuclear transcription factor which may control the basal and inducible expression of over 200 genes. These genes contain antioxidant response elements (AREs) in their regulatory regions by heterodimerizing with small MAF proteins [31]. Kelch-like ECH-associated protein 1 (Keap1) is considered to be a sensor of oxidative stress since its cysteine residue is sensitive to ROS and electrophilic reagents [32]. Keap1 participates in the degradation process of Nrf2 by ubiquitination and suppresses the transcriptional activity of Nrf2. An appropriate interaction between Keap1 and Nrf2 is considered critical for efficient deactivation of Nrf2, but modification of Keap1 thiols by oxidative stress is likely to induce conformational alterations in the overall structure of the Keap1-Nrf2 heteromer. Subsequently, Nrf2 binds the ARE of the associated gene by integrating with MAF. The Keap1-Nrf2-Keap1 complex then activates the expression of the antioxidant gene and initiates the antioxidant defense system [33]. Research of the Keap1-Nrf2 pathway suggests that ROS might be involved in the activation of Nrf2 by stimulating signaling pathways like the MAPK and phosphatidylinositol-4,5-bisphosphate 3-kinase (PI3K) signaling pathways [34, 35]. Degradation of intracellular Nrf2 by ubiquitination is inhibited during oxidative stress. When the redox balance is restored, Nrf2 returns to the cytoplasm for degradation by ubiquitination, thereby returning to its basal level.

ARE is a specific binding sequence in the DNA promoter that is mainly stimulated by Nrf2. The Keap1-Nrf2-ARE pathway is a major mechanism for intracellular antioxidation by regulating type 2 detoxification enzymes, superoxide dismutase (SOD), antioxidases, and GSH synthesis-related enzymes. It has been demonstrated that the Keap1-Nrf2-ARE pathway is involved in the initiation and development of several tumors [36]. ROS is capable of altering the structure and function of DNA and proteins and inducing gene mutations and tumorigenesis. The Keap1-Nrf2-ARE pathway plays a major role in detoxification of exogenous carcinogens [37, 38]. It has been reported that the Nrf2 agonist shows anti-tumor effect in multiple models of tumors, including BCa [39]. Therefore, the Keap1-Nrf2-ARE pathway is an important component of the oxidative defense system.

## ROS/oxidative stress-related mechanism in BCa

The role of ROS in cancer cells is complicated. ROS, mediated by the low valence state of metal ions like Fe^2+^ and Cr^3+^, can cause oxidative damage to DNA. Purines and pyrimidines, located outside of the DNA double helix, are most sensitive to free radicals. Oxidative damage causes gene mutations through basic base modification and chain reactions. A chain reaction can then lead to DNA strand breakage, DNA–DNA crosslink, or DNA–protein crosslink. DNA damage affecting cancer-associated genes, such as Ras and p53, can directly lead to cancer. The Ras gene mutation can be identified in approximately 30% of patients with BCa and the p53 gene mutation is correlated with over 50% of patients with BCa [40, 41]. ROS can undermine the stability of DNA and cellular organelles and facilitate oncogenic transformation of normal cells. Intracellular ROS levels of transformed cells are maintained at a level higher than that of normal cells due to abnormal metabolism. This could contribute to gene mutations involved in cancer initiation [42]. Oxidative stress can also lead to hypermethylation of tumor suppressors and hypomethylation of oncogenes, which implies its role in cancer initiation [43, 44].

Mounting evidences demonstrate that an increase in ROS levels not only participates in the initiation of BCa but also contributes to its invasion and metastasis. ROS is associated with H-Ras-induced oncogenic transformation of multiple tumor types [45–47]. Aberrant expression of the oncogene H-Ras leads to excessive production of ROS in J82 human BCa cells. H-Ras upregulates the expression of NADPH oxidase-1 (NOX-1) through ERK signaling pathway, resulting in increased intracellular ROS levels [48]. The ERK-NOX-1 signaling pathway can also be activated by overexpression of FGFR or EGFR, which leads to increased ROS levels, but its mechanism remains under investigation. Therefore, the primary reasons for elevated ROS levels in BCa cells are due to (1) aberrant expression of the membrane-related growth factor receptor, oncogene H-Ras, and (2) continuous activation of the ERK signaling pathway. Many molecules might collaborate to sense and transduce ROS-related signaling in BCa cells during bladder cancer initiation and progression (Table 1).

**Table 1:** Oxidative stress-associated regulators in tumorigenesis of bladder cancer (BCa)

Stage	Regulator	Expression and effect on ROS	Outcome	Model	References
Initiation	Serum protein acidic and rich in cysteine (SPARC)	Expression↑	Prognosis↓ Survival rate↓	Human bladder cancer tissues	[52]
		SPARC-*KO* → ROS↑	Invasion↑ Metastasis↑	BBN-induced SPARC-*KO* rats	[53]
	Mitochondrial cytochrome b (MT-CYB)	Mutation → ROS↑ → NF-κB↑, cyclin D1↑	Growth↑ Invasion↑ Vascularization↑	Murine xenograft BCa model	[54, 55]
	Leukotriene B4 receptor 2 gene (LTB4R2)	Expression↑ → NOX-1↑, NOX-4↑ → ROS↑, NF-κB↑	Invasion↑ Metastasis↑	Bladder cancer 253 J-BV cells	[56]
Progression	Transmembrane-4-L-six-family-1 (TM4SF1)	Expression↓ → ROS↑	Cell cycle↓ Cell apoptosis↑	Bladder cancer RT-4, 5637, T24, UM-UC-3 cells	[61]
	Alkylated DNA repair protein alkB homolog 8 (ALKBH8)	Expression↓ → ROS↑ → JNK, P38↑	Cell apoptosis↑ Invasion↓ Angiogenesis↓	KU7 cells; orthotopic mouse model	[62]
	Glucose-6-phosphate dehydrogenase (G6PD)	Expression↑	Poor prognosis↑	Human bladder cancer tissues	[63]
		Expression↓ → ROS↑ → protein kinase B↓	Cell viability↓ Cell apoptosis↑	Bladder cancer T24 cells	[63]
	Matrix metalloproteinase-9 (MMP9)/Vascular endothelial growth factor (VEGF)	ROS↑ → MMP9/ VEGF↑	Invasion↑ Vascularization↑	Bladder cancer 253J-BV cells	[64]

↑Up-regulation/activation, ↓ down-regulation/inactivation, *KO* knockout

### Role of SPARC in BCa

Serum protein acidic and rich in cysteine (SPARC) is involved in the initiation of various types of tumors, but its role in BCa remains controversial [49]. SPARC is expressed in the urothelium and the subepithelial matrix of rats and human as well as in cultured human urothelium cells. For the urothelium of humans and rats, SPARC plays a role in anti-adherence and anti-proliferation [50, 51]. It was reported that SPARC is also expressed in BCa tissues, and the overexpression of SPARC is negatively correlated with the prognosis and survival rate of invasive BCa patients [52]. In a recent study, BCa was induced by the chemical carcinogen BBN in SPARC-knockout (KO) rats and wild type (WT) rats. The results showed that SPARC-KO rats had accelerated oncogenic transformation, tumorigenesis, invasion, and a significantly lower survival rate. The production of ROS was elevated in the SPARC-KO group, and this confirmed that the elevation of ROS was positively correlated with the invasion and metastasis of BCa. As a biomarker of oxidative stress, 8-hydroxy-2′-deoxyguanosine (8-OHdG) levels in the SPARC-KO group was obviously higher compared with that in the WT group, which implies that SPARC is mediated by oxidative stress and inflammation. In comparison with the control group, phosphorylation of p38, MAPK, JNK, c-Jun, and p65-NF-κB was much higher in the SPARC-KO group. Furthermore, phosphorylated c-Jun and p65-NF-κB were found in the nucleus of BCa cells in the SPARC-KO group. The proliferation of BCa cells in the SPARC-KO group was much higher than that of primary cultured normal urothelial cells and positively correlated with ROS levels. In addition, supplementing cells with exogenous SPARC is able to partially inhibit the generation of extracellular H_2_O_2_, which suggests that aberrant expression of SPARC may be involved in the initiation of BCa [53].

### Mutation of mitochondrial DNA/mitochondrial cytochrome b in BCa

The mitochondria are the main organelles for energy metabolism and ROS generation. There are 2–10 mitochondrial DNA (mtDNA) in each mitochondrium. Besides genomic DNA, oxidative damages in mtDNA has also been linked to tumorigenesis. Mutations in mtDNA impact oxidative phosphorylation (OXPHOS) of the electron transport chain. Subsequently, ROS is produced during the oxidative phosphorylation of mtDNA, and this causes damage to nuclear DNA, which leads to constitutive oxidative stress and tumorigenesis. Previous studies have shown that, for multiple human tumors, the mitochondrial gene coding complex is mutated and the regional localization of mtDNA is altered. Generally, mtDNA is susceptible to ROS and oxidative damage because of its limited repair capacity and its location adjacent to the respiratory chain. After oxidative damage of mtDNA, the genes that encode the proteins of the respiratory chain and ROS are up-regulated due to dysfunction of the respiratory chain, which creates a vicious cycle. Recently, the role of mitochondria in the development of BCa has raised concerns. It has been reported that there was a correlation between mutant mtDNA and metabolic transformation in BCa [54]. Mutation of the mitochondrial cytochrome b (MT-CYB) gene was identified in a human BCa cell. In rat BCa cells, the expression of mutant MT-CYB led to an increase of ROS and expression of NF-κB and cyclin D1, which promoted the growth, invasion, and vascularization of tumors. After treatment with antioxidant vitamin C, production of ROS was reduced and proliferation of BCa cells was inhibited, which verified that ROS was pivotal for MT-CYB-induced growth [55].

### Role of LTB4R2 in BCa

During the progression of BCa, the expression of the membrane-associated leukotriene B4 receptor 2 gene (LTB4R2) increases. LTB4R2 promotes the expression of NADPH oxidase-1 and -4 (NOX-1 and NOX-4), which leads to the generation of ROS and the activation of NF-κB and further promotes the invasion and metastasis of BCa both in vivo and in vitro. When the expression of NOX-1 and NOX-4 were decreased by RNA interference (RNAi), the production of ROS and the invasion capability of BCa cells were significantly decreased, which implies that NOX-1, NOX-2, and ROS are important for maintaining the malignant properties of BCa cells [56].

### Role of TM4SF1 in BCa

Bioinformatic analysis has been used to identify biomarkers for human disease, including BCa. Bioinformatic analysis of gene expression data has shown that oxidative stress-related genes were significantly associated with the development of BCa [57]. Transmembrane-4-L-six-family-1 (TM4SF1) is a member of the L6 family and functions as a signal transducer to regulate cell development, growth, and motility in many solid malignancies [58–60]. Cao et al. [61] found that TM4SF1 was strongly up-regulated in human muscle invasive bladder cancer (MIBC) tissues and had significant positive correlation (*p* < 0.05) with the T stage, TNM stage, lymph node metastasis status, and the survival rates of MIBC patients. This indicates a positive association between TM4SF1 expression and the poorer prognosis of MIBC patients. Functionally, it has been shown that the knockdown of TM4SF1 induces cell cycle arrest and apoptosis, possibly via the up-regulation of ROS in BCa cells [61]. Moreover, these observations could be reversed by treatment with GW9662 (antagonist of PPARg) and resveratrol (activator of SIRT1), which are known to regulate ROS production. Taken together, their results suggest that a high expression of TM4SF1 predicts a poor prognosis for MIBC [61].

### Role of ALKBH8 in BCa

Newly identified human alkylated DNA repair protein alkB homolog 8 (ALKBH8) is associated with the occurrence of multiple tumors including BCa. In examining the role and function of ALKBH8 in human bladder cancer development in vitro, they found that silencing of ALKBH8 through siRNA transfection reduced ROS production via down-regulation of NAD(P)H oxidase-1 (NOX-1) and induced apoptosis through subsequent activation of c-Jun NH(2)-terminal kinase (JNK) and p38 [62]. Meanwhile, overexpression of NOX-1 and ALKBH8 promoted BCa cell proliferation. The findings verified that ALKBH8, NOX-1, and ROS are key factors for the maintenance and progression of human BCa cells.

### Role of G6PD in BCa

Chen et al. demonstrated that glucose-6-phosphate dehydrogenase (G6PD) was an oncogene in BCa. High G6PD expression was found to be a poor prognostic factor in BCa, and the level of G6PD expression increased as the tumor stage progressed. Knockdown of G6PD suppressed cell viability and increased apoptosis by promoting the accumulation of intracellular ROS and the suppression of protein kinase B pathway [63].

### Role of MMP9/VEGF in BCa

SOD converts O^2−^ into H_2_O_2_ in mitochondria. The level of SOD in normal tissues of bladder was reported to be much higher than that in bladder tumors tissues [64]. Additionally, increased H_2_O_2_ enhances the expression of matrix metalloproteinase-9 (MMP9) and vascular endothelial growth factor (VEGF) which contributes to invasion and vascularization of tumors. In this respect, ROS appeared to be involved in invasion and vascularization of tumors by regulating the expression of MMP9 and VEGF [65].

## ROS-manipulation strategies in bladder cancer treatment

ROS is a double-edged sword. Moderate levels of ROS can sustain or even promote cancer cell survival, whereas excessive levels kill them. The dosage, duration, type, and site of ROS production determine its role as a survival or apoptotic signal. Up-regulation of ROS has been considered anti-oncogenic, since ROS can cause lethal oxidative damage to DNA and trigger cancer cell death. This happens when the level of ROS is beyond the limit of that in cancer cells [66]. Many agents and target genes could alter ROS levels, which leads to cancer cell death (Table 2).

**Table 2:** Therapeutic potential of ROS-targeted strategies in bladder cancer treatment

Drugs or targeted gene	Mechanism of possible function	Model	References
Piperlongumine	Piperlongumine → GSH↓ → ROS↑ → cell apoptosis↑	Bladder cancer EJ, T24 and BIU-87 cells	[66, 68]
Licochalcone A (LCA)	LCA → ROS↑ → cell proliferation ↓	Bladder cancer T24 cells	[69]
Histone deacetylase inhibitor (HDACI)	HDACI → ERK/NOX-1↑ → ROS↑ → cell apoptosis↑	Bladder cancer J82-Ras cells	[70–72]
Sanguinarine	Sanguinarine → ROS↑ → Bax↑, Bid↓, XIAP↓ → cell apoptosis↑	Bladder cancer T24, EJ and 5637 cells	[73]
Compound K (CK)	CK → ROS↑ → p38/MAPK↑ → cell apoptosis↑	Bladder cancer T24 cells	[74]
Vitamin K2	Vitamin K2 → ROS↑ → JNK/p38/MAPK↑ → cell apoptosis↑	Bladder cancer T24, J82, EJ cells	[75]
Altholactone	Altholactone → ROS↑ → p38/MAPK↑, Akt↓ → cell apoptosis↑	Bladder cancer T24 cells	[76]

↑Up-regulation/activation; ↓ down-regulation/inactivation

### Piperlongumine

Piperlongumine, a natural alkaloid isolated from longer pepper plants, may induce apoptosis in cancer cells by increasing ROS generation [67, 68]. Lakshmi et al. [67] reported that piperlongumine could induce apoptosis in cancer cells by inhibiting the activity of GSH and increasing intracellular ROS levels. High-throughput screening was conducted for a number of anti-cancer low-molecular-weight compounds with 6 types of human normal cells and 13 types of cancer cells. The results showed that piperlongumine might selectively kill multiple cancer cells in vitro, and the antineoplastic property was well verified in further animal experiments. Moreover, ROS could also mediate membrane damage with lipid peroxidation by opening mitochondrial membrane permeability transition pore (MPT) which subsequently induces Ca^2+^ outflow, cytochrome C release, and caspase-3 cleavage, resulting in apoptosis. Besides DNA and membrane damage, ROS can kill cancer cells by oxidative cleavage of polypeptide chains. In our study, piperlongumine markedly elevated ROS and the administration of antioxidants abolished piperlongumine-induced cell proliferation inhibition, G2/M phase arrest, and migration suppression in bladder cancer cells [69].

### Licochalcone A

Jiang et al. [70] found that Licochalcone A (LCA) could inhibit the proliferation of human bladder cancer T24 cells by increasing intracellular ROS levels. Their results indicate that LCA inhibits T24 cell proliferation in a concentration-dependent manner, with an IC50 value of approximately 55 μM. The LCA-induced ROS production is inhibited by the co-treatment of LCA and free radical scavenger *N*-acetyl-cysteine (NAC). The ratio of GSH/GSSG also decreased in a concentration-dependent manner. The results suggest that LCA inhibits proliferation by increasing intracellular ROS levels, which then leads to an oxidative stress status in T24 cells.

### Histone deacetylase inhibitor

Histone deacetylase inhibitor (HDACI), a new antineoplastic agent, has the properties of anti-metastasis and anti-vascularization. It has been shown that HDACI selectively induced apoptosis of H-Ras oncogene-expressed human BCa cells J82 (J82-Ras) but not of normal cells [71, 72]. The sensitivity of H-Ras oncogene-expressed J82 cells to HDACI was nearly 100 times higher than that of normal cells [73]. After treatment with HDACI, the intracellular ERK/NOX-1 signaling pathway was triggered in both J82-Ras and J82-WT cells, and ROS production in mitochondria was elevated. The increase in intracellular ROS levels in J82-Ras cells was found to be more significant than that in J82-WT cells. ROS in high concentrations has been shown to stimulate the caspase 8/9/3/7 signaling pathway and result in cell apoptosis.

### Sanguinarine

Han et al. [74] investigated the possible antineoplastic mechanisms of sanguinarine, also known as benzophenanthridine alkaloid, using three cultured human BCa cell lines. Sanguinarine may induce apoptosis; therefore, sanguinarine-treated bladder cancer cells presented growth inhibition in a concentration-dependent manner. Sanguinarine-induced apoptosis was correlated with the up-regulation of Bax, the down-regulation of Bid and XIAP, the activation of caspases (-3, -8, and -9), and the increase of ROS levels. The ROS scavenger NAC completely reversed sanguinarine-induced apoptosis. In addition, sanguinarine effectively increased the activity of JNK and the expression of early growth response gene-1 (Egr-1), which could also be reversed by pretreatment with NAC.

### Compound K and other agents

As a major metabolite of ginsenoside, Compound K (CK) has been shown to exhibit anti-cancer bioactivity. Apoptosis in CK-treated human BCa T24 cells remarkably increases with dosage in a time-dependent manner. CK treatment was shown to induce intracellular ROS accumulation and activate the p38/MAPK signaling pathway in T24 cells. SB203580, an inhibitor of the p38/MAPK signaling pathway, could block CK-induced cell apoptosis. It appears that CK triggers cell apoptosis by inducing excessive ROS production and activation of the p38/MAPK signaling pathway in T24 cells [75]. Duan [76] revealed that vitamin K2 induces apoptosis in bladder cancer cells via ROS-mediated JNK/p38 MAPK pathways, and Zhao [77] demonstrated that altholactone-induced ROS-dependent apoptosis in BCa cells via mitochondrial dysfunction, MAPK-p38 activation, and Akt suppression.

Based on the information summarized above, the use of these agents is very promising. However, due to unsatisfactory selectivity, complicated internal metabolism, and inter-individual differences in drug efficacy, very few agents were evaluated by cancer clinical trials. Only a histone deacetylase inhibitor, chidamide, was evaluated in a phase II study for refractory peripheral T-cell lymphoma [78]. So, the other agents mentioned above require further research and clinical trials before final use.

## Conclusion

Redox balance in mammalian cells is essential for physiological health and redox imbalance promotes various diseases. In spite of that, ROS are involved in many important molecular biological processes by initiation of multiple signaling pathways. Moderate or high levels of intracellular ROS may have comprehensive cellular or molecular effects, including cellular mitosis, growth inhibition, and cellular apoptosis in a concentration-dependent manner. With in-depth studies on oxidative stress-induced tumorigenesis, redox balance has gained interest for its prospective application for clinical trials and experiments in animal models. Further studies of ROS and oxidative stress would be beneficial for a better understanding of tumorigenesis and the design of new targets for treatment.

A number of phytochemicals targeted towards ROS metabolism can selectively kill cancer cells by raising ROS levels beyond a toxic threshold. In comparison with normal cells, cancer cells have higher levels of endogenous ROS. Hence, the toxic threshold can be easily reached in cancer cells. Therefore, intracellular ROS levels can be up-regulated in accordance with this characteristic. On one hand, some agents interrupt maintenance of cancer cell activity by decreasing ROS levels or inhibiting ROS-related signaling pathways. On the other hand, some agents increase intracellular ROS levels up to a lethal threshold, thereby directly killing cancer cells. In brief, changes in ROS levels and an oxidative stress state cast new light on the treatment of bladder cancer. The agents that may specifically induce ROS-mediated apoptosis in cancer cells should be used to develop a highly effective treatment strategy with minimal side effects in BCa patients.

## Abbreviations

ALKBH8: Alkylated DNA repair protein alkB homolog 8
ASK1: Apoptosis signal-regulating kinase 1
BCa: Bladder cancer
Egr-1: Early growth response gene-1
ERK: Extracellular signal-regulated kinase
FGFR3: Fibroblast growth factor receptor 3
G6PD: Glucose-6-phosphate dehydrogenase
HDACI: Histone deacetylase inhibitor
JNK: c-JunN-terminal kinase
LCA: Licochalcone A
LTB4R2: Leukotriene B4 receptor 2 gene
MIBC: Muscle invasive bladder cancer
MKPs: MAP kinase phosphatases
MMP9: Matrix metalloproteinase-9
MT-CYB: Mitochondrial cytochrome b
NRF2: Nuclear factor erythroid 2-related factor 2
ROS: Reactive oxygen species
SPARC: Serum protein acidic and rich in cysteine
TM4SF1: Transmembrane-4-L-six-family-1
VEGF: Vascular endothelial growth factor
